# *ASXL1* mutations are associated with distinct epigenomic alterations that lead to sensitivity to venetoclax and azacytidine

**DOI:** 10.1038/s41408-021-00541-0

**Published:** 2021-09-21

**Authors:** Nora E. Rahmani, Nandini Ramachandra, Srabani Sahu, Nadege Gitego, Andrea Lopez, Kith Pradhan, Tushar D. Bhagat, Shanisha Gordon-Mitchell, Bianca Rivera Pena, Mohammad Kazemi, Keshav Rao, Orsi Giricz, Shahina Bano Maqbool, Raul Olea, Yongmei Zhao, Jinghang Zhang, Hamid Dolatshad, Vickram Tittrea, Dharamveer Tatwavedi, Shalini Singh, Juseong Lee, Tianyu Sun, Ulrich Steidl, Aditi Shastri, Daichi Inoue, Omar Abdel-Wahab, Andrea Pellagatti, Evripidis Gavathiotis, Jacqueline Boultwood, Amit Verma

**Affiliations:** 1 Department of Oncology, Albert Einstein College of Medicine, Montefiore Medical Center, Bronx, NY USA; 2 Blood Cancer UK Molecular Haematology Unit, Nuffield Division of Clinical Laboratory Sciences, Radcliffe Department of Medicine, University of Oxford, Oxford, UK; 3 Department of Biochemistry, Albert Einstein College of Medicine, Montefiore Medical Center, Bronx, NY USA; 4 Department of Genetics, Albert Einstein College of Medicine, Montefiore Medical Center, Bronx, NY USA; 5 Department of Microbiology and Immunology, Albert Einstein College of Medicine, Montefiore Medical Center, Bronx, NY USA; 6 Department of Medicine, Division of Hemato-Oncology, Montefiore Medical Center, Albert Einstein Cancer Center, Bronx, NY USA; 7 Department of Developmental and Molecular Biology, Montefiore Medical Center, Albert Einstein Cancer Center, Bronx, NY USA; 8 Human Oncology and Pathogenesis Program, Memorial Sloan Kettering Cancer Center, New York, NY USA; 9 NIHR Oxford Biomedical Research Centre Haematology Theme, Oxford, UK

**Keywords:** Myelodysplastic syndrome, Acute myeloid leukaemia

## Abstract

The BCL2-inhibitor, Venetoclax (VEN), has shown significant anti-leukemic efficacy in combination with the DNMT-inhibitor, Azacytidine (AZA). To explore the mechanisms underlying the selective sensitivity of mutant leukemia cells to VEN and AZA, we used cell-based isogenic models containing a common leukemia-associated mutation in the epigenetic regulator *ASXL1*. KBM5 cells with CRISPR/Cas9-mediated correction of the *ASXL1*^*G710X*^ mutation showed reduced leukemic growth, increased myeloid differentiation, and decreased *HOXA* and *BCL2* gene expression in vitro compared to uncorrected KBM5 cells. Increased expression of the anti-apoptotic gene, *BCL2*, was also observed in bone marrow CD34+ cells from *ASXL1* mutant MDS patients compared to CD34+ cells from wild-type MDS cases. ATAC-sequencing demonstrated open chromatin at the *BCL2* promoter in the ASXL1 mutant KBM5 cells. BH3 profiling demonstrated increased dependence of mutant cells on *BCL2*. Upon treatment with VEN, mutant cells demonstrated increased growth inhibition. In addition, genome-wide methylome analysis of primary MDS samples and isogenic cell lines demonstrated increased gene-body methylation in *ASXL1* mutant cells, with consequently increased sensitivity to AZA. These data mechanistically link the common leukemia-associated mutation *ASXL1* to enhanced sensitivity to VEN and AZA via epigenetic upregulation of *BCL2* expression and widespread alterations in DNA methylation.

## Introduction

Myelodysplastic syndromes (MDS) and acute myeloid leukemia (AML) are generally incurable malignancies characterized by high rates of chemo-refractoriness and intrinsic resistance to apoptosis. Studies have demonstrated overexpression of antiapoptotic proteins, such as BCL2, in MDS/AML [1–3], although the mechanistic basis of this upregulation is not well understood. This becomes important as Venetoclax (VEN), a small molecule inhibitor of *BCL2*, has shown efficacy in conjunction with DNMT-inhibitors, such as Azacytidine (AZA) [1, 4–6], resulting in high rates of complete remission [1, 4]. As the selectivity of malignant cells to this combination therapy is not well elucidated, we used a cellular model of a common leukemia-associated mutation in the epigenetic regulator *ASXL1* to determine the expression levels of *BCL2* and investigate the mechanistic basis of *BCL2* overexpression to determine whether this regulates increased sensitivity to VEN and AZA.

*ASXL1*, additional sex comb-like 1, is recurrently mutated in a range of myeloid malignancies, including MDS, AML, and chronic myelomonocytic leukemia, and has been noted to be one of the most commonly occurring mutations in patients with clonal hematopoiesis and MDS [7–9]. *ASXL1* mutations are usually heterozygous [10], most frequently occur at G646 [10], and are associated with inferior overall survival [10, 11]. *ASXL1* is known to play a central role in epigenetic regulation, by activating or repressing the transcription of genes implicated in differentiation and proliferation through its effects on histone methylation [11, 12]. In particular, *ASXL1* is thought to be involved in the recruitment of the Polycomb repressive complex 2 (PRC2) to specific loci, including the HOXA cluster [11, 12]. As the PRC2 acts through epigenetic modification of chromatin to maintain genes in a repressed state, an inactivating *ASXL1* mutation leads to reduced PRC2 activity resulting in aberrant activation of genes [11, 12].

We previously determined the downstream effects of the *ASXL1* mutation using the KBM5 chronic myelogenous leukemia (CML) cell line, that carries a homozygous nonsense *ASXL1*^*G710X*^ mutation, resulting in loss of ASXL1 protein expression [12]. We observed that the *ASXL1* mutation results in the failure of the PRC2 complex to recruit the HOXA cluster, leading to increased expression of *HOXA* genes and resultant myeloid transformation. Through CRISPR/Cas9 gene-editing technology, we corrected the *ASXL1*^*G710X*^ mutation in KBM5 cells, resulting in restored ASXL1 protein expression. In particular, we showed that the correction of the *ASXL1* mutation restored the function of PRC2, including *HOXA* gene down-regulation with resultant reduction in cell growth. Although DNA methylation is critically important in regulating gene expression in hematopoiesis, the effect of the *ASXL1* mutation on cytosine methylation is not well described. Therefore, in our current study, we used the *ASXL1*^*G710X*^ mutant KBM5 CML cell line and the CRISPR/Cas9 homozygous corrected isogenic cells to evaluate the effects of the *ASXL1* mutation on gene expression, chromatin accessibility, and cytosine methylation, and to correlate these findings to functional responses to VEN and AZA.

## Materials and methods

### Cell lines and culture conditions

KBM5 cells derived from a CML patient in blast crisis were previously provided by Dr. Bing Z. Carter, MD Anderson Cancer Center. CRISPR–Cas9 correction of the *ASXL1* mutation in the *ASXL1* mutant KBM5 cells was conducted using the pX458 vector (Addgene) expressing Cas9 and containing a cloning site for the single guide RNA (sgRNA) sequence, as previously described [12]. Sanger sequencing was used to confirm the correct insertion of the sgRNA sequences. *ASXL1* mutant KBM5 cells and the isogenic homozygous ASXL1 corrected cells [12] were maintained in Iscove’s Modified Dulbecco’s medium containing 10% heat-inactivated fetal bovine serum, 1% l-glutamine, 100 units/mL penicillin, and 100 μg/mL streptomycin.

### Cell viability assay

Cell viability analysis was performed as previously described [13]. In brief, isogenic KBM5 cells were seeded in 96-well plates with various concentrations of Azacytidine for 24–96 h or Venetoclax for 48 h. Details are provided in the Supplementary Methods.

### Flow cytometry analysis for myeloid differentiation markers and apoptosis

*Myeloid differentiation*: *ASXL1* mutant and corrected KBM5 cells were stained with Human CD11b–APC Conjugate (Thermo Fisher Scientific, Waltham, MA, USA, Cat No. CD11B05, clone VIM12), Human CD14-Pacific Blue^TM^ (Thermo Fisher Scientific, Cat No. MHCD1428, clone TuK4), and Anti-Human CD15-PerCP-eFluor^TM^710 (Thermo Fisher Scientific, Cat No. 46-0158-42, clone MMA). Data acquisition was performed using a BD FACS LSRII instrument (BD Biosciences, Franklin Lakes, NJ, USA) and data were analyzed using FlowJo software version 10.6.1 (BD Biosciences). *Apoptosis*: *ASXL1* mutant and corrected KBM5 cells were treated with 40 nM of VEN for 48 h with controls. At hour 48, cells were stained with FITC-conjugated Annexin V Alexa Fluor 488 (Thermo Fisher Scientific, Cat No. A13199) and Propidium iodide, and flow cytometry was conducted using a BD FACS LSRII instrument to evaluate the percentage of viable cells, the percentage of cells in early and late apoptosis, and cells undergoing apoptosis. Data were analyzed using FlowJo.

### DNA extraction

Genomic DNA was extracted from the *ASXL1* mutant and corrected KBM5 cells using the Blood & Cell Culture DNA Mini Kit (Qiagen, Germantown, MD, USA, Cat No./ID: 13323), following the manufacturer’s protocol.

### RNA-sequencing

Total RNA was isolated from the *ASXL1* mutant and the corrected KBM5 cells using the Qiagen RNeasy Mini Kit (Cat No./ID: 74104) and the integrity of the total RNASeq libraries were multiplexed and sequenced as 1×150 bp single end on NEXTSEQ 500 (Illumina, San Diego, CA, USA) following standard protocols. Sequencing data was QC and analyzed using GSNAP (http://research-pub.gene.com/gmap/) software for mapping to a reference genome and HTSeq-count (http://www-huber.embl.de/users/anders/HTSeq/) to assign unique counts to ENSEMBL annotated transcripts. The Bioconductor package *DESeq* (http://bioconductor.org) was used to determine the relative counts of sequences from each gene relative to each other (transcriptional profiling) and for normalization and statistical comparison.

### ATAC-sequencing

A suspension of 50 000 cells from both the *ASXL1* mutant and the corrected KBM5 cells were harvested, resuspended in cold ATAC-seq resuspension buffer (RSB), and subjected to Omni-ATAC-seq [14]. ATAC-Seq libraries were QC and multiplexed for sequencing as 2 × 75 bp paired-end on NEXTSEQ 500 (Illumina) following standard protocols. Sequencing data was QC and analyzed using Bowtie 2 (version 2.2.3) with parameters allowing for soft clipping to align the sequencing reads for each sample to the NCBI reference human genome sequence (Build 37/hg19). Peak calling for each sample was performed using MACS2 (version 2.1.0) with default parameters. For discovering differential peaks between samples, one sample was treated as the target, the other as the background control.

### Methylation DNA Immunoprecipitation

Methylation DNA Immunoprecipitation (MeDIP)-sequencing was performed using 20 μg of purified genomic DNA from each sample as per Active Motif’s MeDIP Kit (Carlsbad, CA, USA, Cat No. 55009, Version B2) and NEBNext Ultra II DNA Library Prep Kit for Illumina (Cat No. E7645S), following the manufacturer’s protocol. MeDIP-Seq libraries were QC and multiplexed for sequencing as 2 × 150 bp paired-end on NEXTSEQ 500 (Illumina) following standard protocols. Sequencing data was QC and analyzed as follows: Fastq files were trimmed of their adapters with “trim_galore”, then aligned to the GRCh38 reference with “bowtie2”. Duplicate reads were marked with “Picard tools” and peaks were found with “MACS2” software under default parameter settings.

### Quantitative real-time PCR

*ASXL1* mutant and corrected KBM5 cells were harvested and total RNA was extracted using the RNeasy Mini Kit (Qiagen, Cat No./ID: 74104). qRT-PCR was performed for various genes as described in the Supplementary Methods.

### BH3 Profiling

Cells were plated at a density of 2 × 10^4^ cells/well in MEB buffer (150 mM mannitol, 10 mM HEPES-KOH pH 7.5, 50 mM KCl, 0.02 mM EGTA, 0.02 mM EDTA, 0.1% BSA, 5 mM Succinate). BIM, BID, PUMA BMF-y, BAD, HRK-y, FS1 and MS1 BH3 peptides at indicated concentrations; Puma2A peptide (final concentration of 25 μM); alamethicin (final concentration of 25 μM); CCCP (final concentration of 10 μM) were added to JC1-MEB staining solution (20 ug/mL oligomycin, 20 ug/mL digitonin, 2 μM JC-1, 10 μM 2-mercaptoethanol in MEB) in a black 384-well plate (Corning, Corning, NY, USA, CLS 3573). Single-cell suspensions were washed 2× in PBS and resuspended in MEB at 4× their final density. One volume of the 4× cell suspension was added to one volume of the JCI-MEB staining solution. This 2× cell/staining solution stood at RT in the dark for 10 min to allow cell permeabilization and dye equilibration. A total of 15 μL of the 2× cell/staining solution mix was then added to each treatment well of the plate. The fluorescence was measured at 590 nm emission 545 nM excitation using the M1000 microplate reader (TECAN) at 30 °C every 15 min for a total of 3 h. Percentage of depolarization was calculated by normalization to the AUC of solvent-only control DMSO (0% depolarization) and the positive control CCCP (100% depolarization), as previously described [15]. Bar graph represents the % of mitochondria depolarization of cells detected by JC-1 upon treatment of BH3-derived peptides, *n* = 3.

### DNA methylation analysis by HELP

Peripheral blood and bone marrow mononuclear cells were obtained from MDS patients with or without *ASXL1* mutations. Samples were obtained with informed consent and collected with the approval of the relevant research ethics committees. The HELP assay was performed on DNA extracted from these samples as previously described [16].

### Giemsa staining

Cells were cytospun on slides and stained with Giemsa solution.

### Western blot analysis

*ASXL1* mutant and corrected KBM5 cells (1.2 × 10^7^ cells) were harvested and protein lysates were prepared by incubation for 30 min with western lysis buffer, containing cocktail phosphatase inhibitors and proteases. Immunoblotting with various antibodies was performed by LI-COR western blotting. Details are provided in the Supplementary Methods.

### Statistical analysis

Statistical analysis was performed using GraphPad Prism Version 8 (GraphPad Software, Inc., San Diego, CA, USA). Differences in gene expression (quantitative real-time PCR, cell viability assays, flow cytometry) were tested using a two-sided paired *t* test and two-way ANOVA.

## Results

### CRISPR/Cas9-mediated correction of the *ASXL1* mutation leads to increased myeloid differentiation

**Fig. 1:**
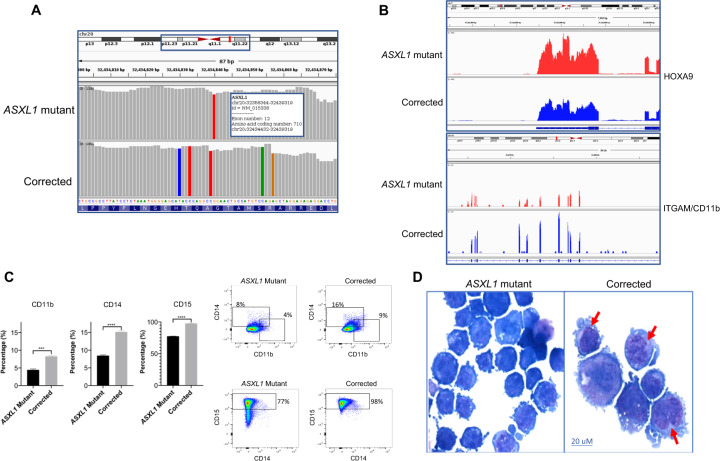
CRISPR/Cas9-mediated correction of the *ASXL1* mutation leads to increased myeloid differentiation. **A** The *ASXL1* gene is made up of 12 exons with the *ASXL1*^G710X^ mutation in KBM5 cells located in exon 12. RNA-sequencing demonstrates CRISPR/Cas9-mediated correction of the *ASXL1*^G710X^ mutation in the leukemic KBM5 cell line. For the *ASXL1* mutant cells, the red bar demonstrates the presence of the *ASXL1* mutation at amino acid location 710, exon 12, chromosome 20. For the corrected cells, the red bar is absent at amino acid location 710, exon 12, chromosome 20, demonstrating correction of the *ASXL1* mutation. In addition, the different colored bars in the corrected trace demonstrate silent nucleotide changes that were introduced to avoid undesired Cas9 activity in the mutation-corrected cells. **B** RNA-sequencing demonstrates that correction of the *ASXL1*^G710X^ mutation in the KBM5 cells by CRISPR/Cas9 gene-editing results in decreased expression of the *HOXA9* gene. Increased expression of *ITGAM/CD11b* is demonstrated in the corrected isogenic cell line, suggesting increased myeloid differentiation. **C﻿** Flow cytometry demonstrates increased expression of three myeloid surface markers, CD11b, CD14, and CD15, in the corrected cell line compared to the *ASXL1* mutant cells, demonstrating increased myeloid differentiation with correction of the *ASXL1*^G710X^ mutation. Representative plots are shown. **D** Representative images of Giemsa stained *ASXL1* mutant cells and those with homozygous correction of the *ASXL1*^G710X^ mutation. Red arrows identify cells with decreased nucleus to cytoplasm ratio and condensed nuclei, consistent with myeloid maturation/differentiation.

CRISPR/Cas9 mediated correction of the *ASXL1*^*G710X*^ mutation in the KBM5 cell line [12] was confirmed by transcriptomic analysis. The RNA-sequencing data demonstrated the presence of the homozygous *ASXL1*^*G710X*^ mutation in the parental KBM5 cell line and the homozygous correction of the mutation in the CRISPR/Cas9-mediated corrected cells (clone G25F7, Fig. 1A). Further evaluation of the RNA-sequencing data demonstrated that as expected, the correction of the *ASXL1*^*G710X*^ mutation was associated with decreased expression of the *HOXA9* gene (Fig. 1B). This is consistent with previous results demonstrating that the *ASXL1* mutation results in failure of the PRC2 complex to recruit the HOXA cluster, resulting in increased expression of *HOXA* genes [11, 12]. Correction of the mutation resulted in increased expression of *ITGAM/CD11b* (Fig. 1B) suggesting myeloid differentiation, and this was further confirmed by flow cytometry demonstrating increased expression of various myeloid differentiation markers, namely ITGAM/CD11b, CD14, and FUT4/CD15 (Fig. 1C, Supplementary Fig. 1). Cytomorphology of *ASXL1* mutant and corrected KBM5 cells demonstrated an increase in myeloid differentiation in the corrected cells with decreased nucleus to cytoplasm ratio, increased vacuolation, and condensed nuclei (Fig. 1D). Furthermore, cell cycle analysis demonstrated reduced S and G2/M cycling in the corrected cells (Supplementary Fig. 2).

### *ASXL1* mutation leads to increased chromatin accessibility and increased expression of *BCL2*

**Fig. 2:**
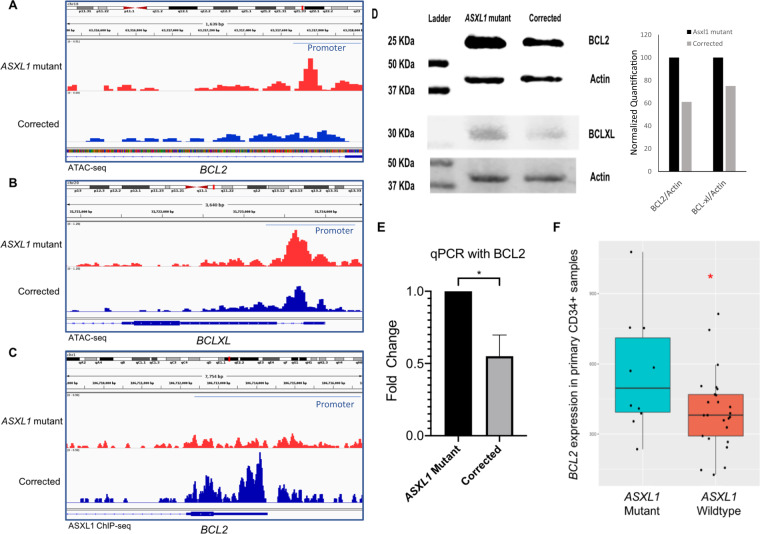
*ASXL1* mutation leads to increased chromatin accessibility and increased expression of *BCL2*. **A**, **B** ATAC-sequencing demonstrates increased chromatin accessibility in the promoter regions of anti-apoptotic genes *BCL2* and *BCLXL* in the mutant *ASXL1* cells compared to the homozygous corrected cells. **C** ChIP-sequencing from a mouse model of *ASXL1* mutant knock-in mice demonstrates wild-type *ASXL1* to be present at the *BCL2* promoter region with no binding of the *ASXL1* mutant seen at this locus. **D** Immunoblotting shows increased protein levels of BCL2 and BCLXL in *ASXL1* mutant cells. The normalized intensity of BCL2/actin is shown as bar graphs. **E** qRT-PCR demonstrates increased expression of BCL2 in *ASXL1* mutant cells. **F** Gene expression analysis of the anti-apoptotic gene *BCL2* in the *ASXL1* mutant and wild-type primary CD34+ patient MDS samples demonstrates significantly higher expression of *BCL2* in the mutant samples compared to the wild-type samples.

To evaluate the effects of the *ASXL1* mutation on genome-wide chromatin accessibility, we conducted ATAC-sequencing on the *ASXL1* mutant and the corrected KBM5 cells. We observed widespread alterations resulting in increased accessibility in important pathways, including cell cycle, cell growth, and cell development pathways in the mutant cells (Supplementary Table 1). Evaluation of specific genes revealed higher ATAC-seq peaks denoting increased chromatin accessibility around the anti-apoptotic genes *BCL2* and *BCLXL* in the *ASXL1* mutant cells when compared to corrected cells (Fig. 2A, B). Further analysis of whether ASXL1 binds to the promoter of BCL2 was conducted with ChIP-sequencing [17]. Analysis of ChIP-seq data from a mouse model of *ASXL1* mutant knock-in demonstrated that wild-type A*SXL1* was found to be present at the *BCL2* promoter, while no binding of the *ASXL1* mutant was seen at this locus (Fig. 2C). These data suggest that *ASXL1* mutation leads to depression of the *BCL2* locus through an inability to bind at its promoter. *BCL2* was found to be elevated at both protein (Fig. 2D) and mRNA levels (Fig. 2E) in the *ASXL1* mutant cells. We evaluated the expression of *BCL2* in bone marrow CD34+ cells from a cohort of MDS and chronic myelomonocytic leukemia patients with and without *ASXL1* mutations [7]. Compared to wild-type samples, we observed significant overexpression of *BCL2* in the *ASXL1* mutant samples, confirming our observations in the isogenic KBM5 cells (Fig. 2F).

### *ASXL1* mutation leads to dependency on *BCL2* and increases sensitivity to the BCL2 inhibitor Venetoclax

**Fig. 3:**
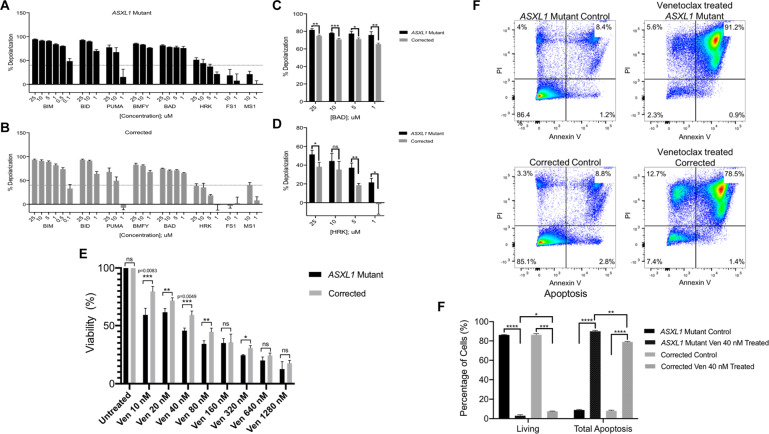
*ASXL1* mutation leads to dependency on *BCL2* and increases sensitivity to BCL2 inhibitor Veneteoclax. **A**, **B** BH3 profiling was conducted to predict the survival dependency of cell lines on antiapoptotic proteins (BCL2, BCL-XL, and MCL-1). Using JC1-BH3 profiling, we assessed depolarization of the *ASXL1* mutant cells and the homozygous corrected cells, upon treatment with BCL-2 family peptides at shown concentrations. Among the selective sensitizers, the BAD peptide led to the highest depolarization, suggesting dependency on BCL2, BCL-XL, or BCL-W. Treatment with HRK, a BCL-XL specific peptide led to depolarization, revealing additional contribution by BCL-XL on the anti-apoptosis effects observed in *ASXL1* mutant cells. **C**, **D** While both cell lines demonstrated depolarization with BAD and HRK, there was significantly more depolarization in the *ASXL1* mutant cells compared to the homozygous corrected cells. **E** In the setting of increased expression of the anti-apoptotic gene *BCL2* in the *ASXL1* mutant cells, we treated the isogenic KBM5 cells with Venetoclax, a BCL2-inhibitor. Cells were treated with a range of doses for 48 h. While both cell lines demonstrated decreased viability, there was significantly more apoptosis in the *ASXL1* mutant cells compared to the homozygous corrected cells. **F** Flow cytometric analysis of apoptosis in *ASXL1* mutant and corrected cells that were either left untreated or treated with Venetoclax 40 nM for 48 h. Treatment with Venetoclax resulted in significantly fewer live cells and more total apoptosis in the Venetoclax treated *ASXL1* mutant cells compared to the Venetoclax treated corrected cells.

BH3 profiling of the *ASXL1* mutant and corrected KBM5 cells was conducted to determine whether increased *BCL2* expression in the *ASXL1* mutant cells leads to greater functional dependence on this anti-apoptotic protein. We observed that among the selective sensitizers, treatment with HRK peptide, a BCL-XL specific peptide, led to higher depolarization in *ASXL1* mutant cells compared to corrected cells, suggesting a dependency on BCL-XL (Fig. 3A, B). Further treatment with the BAD peptide, which potently binds BCL-2 and BCL-XL, showed higher depolarization in *ASXL1* mutant cells, suggesting additional dependency on BCL2 (Fig. 3A, B). Collectively, both cell lines demonstrated depolarization with BAD and HRK peptides, but there was significantly more depolarization in the *ASXL1* mutant cells compared to the corrected cells (Fig. 3C, D).

In the setting of increased expression of and dependence on *BCL2* in the *ASXL1* mutant KBM5 cells, we treated the *ASXL1* mutant and corrected KBM5 cells with VEN. The *ASXL1* mutant cells demonstrated significantly more growth arrest when treated with VEN at a range of doses for 48 h, compared to the corrected cells (Fig. 3E), with the most significant effect noted when treated with 40 nM of VEN. Treatment with 40 nM of VEN for 48 h also resulted in significantly more apoptosis in the A*SXL1* mutant cells compared to the corrected cells (Fig. 3F).

### *ASXL1* mutation leads to aberrant genome methylation patterns

**Fig. 4:**
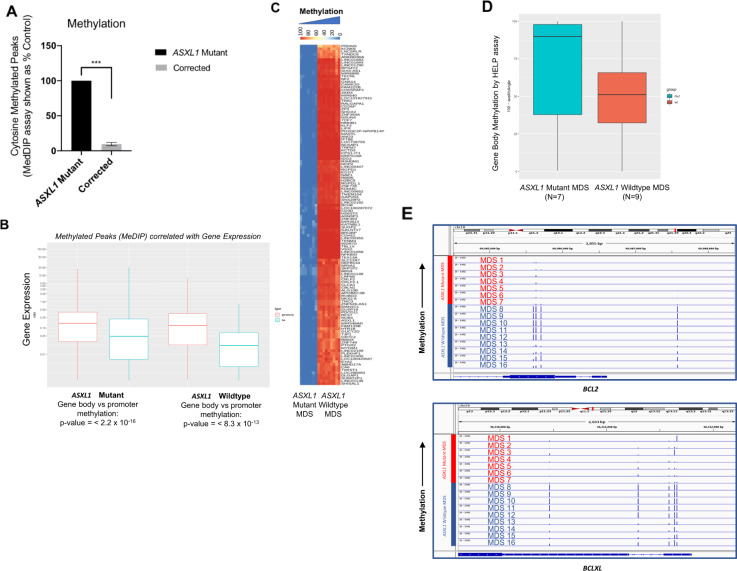
*ASXL1* mutation leads to aberrant genome methylation patterns. **A** Methylation DNA immunoprecipitation demonstrates widespread increased cytosine methylation in the *ASXL1* mutant cells compared to the homozygous corrected cells. Analysis of this data shown as percent control demonstrates significantly more (*p*-value = 0.0003, *t* test) cytosine methylation in the *ASXL1* mutant cells compared to the corrected cells. **B** Evaluation of the methylation DNA immunoprecipitation data alongside the RNA sequencing data demonstrates a clear difference in expression between genes with methylation peaks in the gene bodies versus those with peaks in the gene promoter. Specifically, increased methylation of the gene body was associated with increased gene expression, while increased methylation of the promoter region of genes was associated with decreased gene expression. **C** To validate the results in the primary samples, DNA methylation in a cohort of MDS *ASXL1* mutant and wild-type peripheral blood mononuclear samples was assessed. The HELP assay revealed increased methylation in *ASXL1* mutant samples particularly in the gene bodies, consistent with the results seen in the isogenic cell lines. **D** Genome-wide analysis of *ASXL1* mutant and wild-type patient samples for gene body methylation demonstrate lower average methylation angles among the *ASXL1* mutant samples (*n* = 7) compared to the wild-type samples (*n* = 9), indicating increased methylation in the gene body of the *ASXL1* mutant patient samples, consistent with the results seen in the isogenic cell lines. **﻿E** IGV plots for the *BCL2* and *BCLXL* promoter regions of the *ASXL1* mutant and wild-type patient samples demonstrate hypomethylation in the *ASXL1* mutant samples (MDS 1–7, *n* = 7) compared to the wild type samples (MDS 8–16, *n* = 9).

While it has previously been shown that *ASXL1* may play a role in directly controlling histone methylation via PRC2 interactions [11], the effect of the *ASXL1* mutation on cytosine methylation is not well elucidated. Using the Methylation DNA immunoprecipitation (MeDIP) assay, we studied the effects of the *ASXL1* mutation on DNA methylation, observing a significantly increased number of methylated peaks in the *ASXL1* mutant KBM5 cells compared to the corrected cells (Fig. 4A). The majority of DNA methylation was seen in the gene bodies (Fig. 4B). While this pattern was observed in both the mutant and corrected cells, the difference between gene body and promoter methylation in the mutant cells was significantly greater (Fig. 4B). Integration of the MeDIP data with RNA sequencing data demonstrated that increased methylation of the gene body was associated with increased gene expression, while promoter methylation was associated with decreased gene expression, consistent with other reports [18].

To validate the results in primary patient samples, we assessed DNA methylation in a cohort of MDS *ASXL1* mutant (*n* = 7) and wild-type (*n* = 9) cases. Genome-wide analysis by the HELP assay demonstrated increased methylation in the *ASXL1* mutant samples compared with the wild-type samples (Fig. 4C), particularly in the gene bodies (Fig. 4D), consistent with the results seen in the isogenic KBM5 cell lines. Evaluation of the *BCL2* and *BCLXL* promoter regions of the patient samples revealed hypomethylation in the *ASXL1* mutant samples (MDS 1–7) compared to the wild-type samples (MDS 8–16) (Fig. 4E), consistent with the overexpression of *BCL2* seen in *ASXL1* mutant samples.

### Azacytidine leads to increased differentiation in *ASXL1* mutant cells

**Fig. 5:**
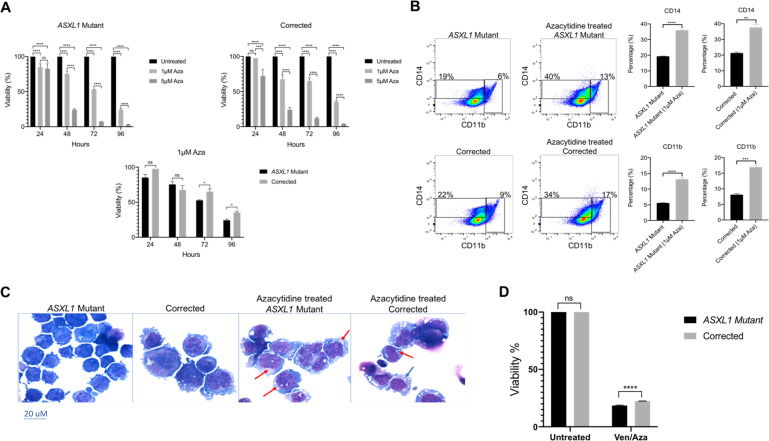
Azacytidine leads to increased differentiation in *ASXL1* mutant cells. **A** Due to the increased methylation in the *ASXL1* mutant cells, we treated the isogenic KBM5 cells with the DNMT inhibitor 5-azacytidine. Proliferation assays of the *ASXL1* mutant cells and homozygous corrected cells treated with 5-Azacytidine revealed significantly decreased cell viability of the *ASXL1* mutant cells, particularly when treated with 1 μM at 72 and 96 h. **B** Flow cytometric analysis of the isogenic KBM5 cells treated with 1 μM 5-Azacytidine for 96 h demonstrated increased differentiation with increased expression of CD11b and CD14 in the treated vs. untreated *ASXL1* mutant cells. Furthermore, there is increased expression of CD11b and CD14 in the treated *ASXL1* mutant cells compared to the untreated homozygous corrected cells. **C** Representative images of Giemsa stained untreated and 5-Azacytidine treated *ASXL1* mutant cells and untreated and 5-Azacytidine treated homozygous corrected cells. Red arrows identify cells with decreased nucleus to cytoplasm ratio and condensed nuclei, consistent with myeloid maturation/differentiation. **D** Proliferation assays of the *ASXL1* mutant and homozygous corrected cells treated with the combination of Venetoclax and 5-Azacytidine revealed significantly decreased cell viability of the *ASXL1* mutant cells.

As DNMT inhibitors show efficacy in MDS/AML, given the increased global methylation we observed in the *ASXL1* mutant KBM5 cells, we next treated the *ASXL1* mutant and corrected cells with the DNMT inhibitor AZA. Treatment with AZA resulted in significantly decreased cell viability of the *ASXL1* mutant cells compared to the corrected cells, particularly when treated at the lower dose of 1 μM at 72 and 96 h (Fig. 5A). Flow cytometric analysis demonstrated increased differentiation with significantly increased expression of the myeloid surface markers CD11b and CD14 in the AZA treated versus untreated *ASXL1* mutant cells (Fig. 5B, Supplementary Fig. 3). Cytomorphology of AZA treated and untreated *ASXL1* mutant and corrected cells demonstrated decreased nucleus to cytoplasm ratio, increased vacuolation, and condensed nuclei in the AZA treated mutant cells, consistent with increased myeloid differentiation (Fig. 5C). Further treatment of the *ASXL1* mutant and corrected cells with the combination treatment of VEN and AZA demonstrated significantly decreased cell viability of the *ASXL1* mutant cells compared to the corrected cells, consistent with the proliferation assays conducted with VEN and AZA alone (Fig. 5D).

## Discussion

While the standard initial treatment for patients with AML is intensive chemotherapy, hypomethylating agents, such as Azacytidine and Decitabine, have been a cornerstone of therapy for older patients with AML and MDS who cannot tolerate intensive induction chemotherapy due to comorbidities. Despite their widespread use, hypomethylating agents as monotherapy often are characterized by a relapse of disease [19]. Venetoclax, a selective BCL2-inhibitor, was initially approved by the US Food and Drug Administration (FDA) for chronic lymphocytic leukemia. More recently in 2018, Venetoclax has been approved for use in combination with a hypomethylating agent, such as Azacytidine or Decitabine, or low-dose cytarabine for upfront therapy of AML in older patients or those unable to tolerate induction chemotherapy. As a single agent, Venetoclax had modest activity in patients with relapsed and refractory AML [1, 19]. However, Venetoclax in combination with a hypomethylating agent, such as Azacytidine or Decitabine, demonstrated significant activity as upfront therapy [19], rapid response, and reassuring lengths of remission [4]. Although the efficacy of Venetoclax in combination with a hypomethylating agent for patients with AML has been established, the mechanism is not clear.

Our study shows that leukemic cells with *ASXL1* mutation are associated with overexpression of *BCL2* leading to dependence on this anti-apoptotic protein in functional assays and increased sensitivity to VEN in vitro. We further show that *ASXL1* mutant cells are also characterized by increased levels of global cytosine methylation and show increased sensitivity to AZA in vitro. These data demonstrate mechanisms for increased efficacy of *ASXL1* mutant leukemia cells to VEN and AZA. Of note, the cells used in our study included those with the leukemic transformation from CML as well as high-risk MDS patients. Other studies have also evaluated correlates for responsiveness to VEN and AZA in leukemia and MDS models [4, 19].

We have previously demonstrated through the use of the *ASXL1*^*G710X*^ mutant KBM5 CML cell line and the CRISPR/Cas9 homozygous corrected isogenic cells, that in the *ASXL1* mutant cells, there was the loss of function of PRC2, resulting in *HOXA* gene over-expression and resultant myeloid transformation [12]. In conjunction with our current findings, we propose that in the presence of the *ASXL1* mutation, the PRC2 complex fails to be recruited to certain loci, in particular *BCL2*, resulting in increased chromatin accessibility leading to increased gene expression and resultant sensitivity to VEN. In contrast, when the *ASXL1* mutation is corrected leading to wildtype ASXL1 protein formation, the PRC2 complex is able to be recruited to specific loci, such as *BCL2*, resulting in decreased chromatin accessibility, with decreased gene expression and decreased sensitivity to VEN.

Furthermore, our studies provide genome-wide maps of chromatin accessibility and DNA methylation in isogenic cell lines and methylome data in primary MDS samples with and without *ASXL1* mutation. These data demonstrate that *ASXL1* mutant cells have widespread alterations in DNA methylation that are predominantly seen in gene bodies and are associated with increased sensitivity to AZA. Taken together, these data support a mechanism in which a common leukemia-associated mutation, *ASXL1*, is linked to epigenetic upregulation of *BCL2* expression and to enhanced sensitivity to VEN and AZA.

## Supplementary information

Supplementary Information Text Summary and Types of Files

Supplementary Information
