# Supplementary material for: ASXL1 mutations are associated with distinct epigenomic alterations that lead to sensitivity to venetoclax and azacytidine
Source: Blood Cancer J. 2021 Sep 21;11(9):157. doi: 10.1038/s41408-021-00541-0 (PMC8455571; doi:10.1038/s41408-021-00541-0)
Supplement: Supplementary file 1 — Supplementary Information Text Summary and Types of Files [file 41408_2021_541_MOESM1_ESM.pdf]

In the Supplementary Information file you will find the following:

- Supplementary Methods
- Supplementary Table 1
- Supplementary Figures 1-3

The Supplementary Information file is submitted as a PDF.
