# Supplementary material for: ASXL1 mutations are associated with distinct epigenomic alterations that lead to sensitivity to venetoclax and azacytidine
Source: Blood Cancer J. 2021 Sep 21;11(9):157. doi: 10.1038/s41408-021-00541-0 (PMC8455571; doi:10.1038/s41408-021-00541-0)
Supplement: Supplementary file 2 — Supplementary Information [file 41408_2021_541_MOESM2_ESM.pdf]

Supplementary Information:

Materials and Methods:

Cell Viability Assay: To analyze the effects of AZA on cell viability, 96-well plates were seeded with ASXL1 mutant and corrected KBM5 cells at a density of 10 000 cells/well and treated with 1 µM or 5 µM of AZA daily on days 0-4 (0-72 hours). Viability was assessed by the addition of Cell Titer Blue (Promega, Madison, WI, USA) on day 5 (96 hours). Fluorescence was measured using the Fluostar Omega Microplate reader (BMG Labtech, Cary, NC, USA). To analyze the effects of VEN on apoptosis and growth arrest, 96-well plates were seeded with ASXL1 mutant and corrected KBM5 cells at a density of 10 000 cells/well and treated on day 0 (0 hours) at 50% dose reductions with concentrations ranging from 1280 nM to 10 nM. Viability was assessed by the addition of Cell Titer Blue (Promega) on day 2 (48 hours). Fluorescence was measured using the Fluostar Omega Microplate reader (BMG Labtech).

Quantitative real-time PCR: Total RNA was reverse transcribed using the Superscript Vilo cDNA synthesis kit (Thermo Fisher Scientific, Cat No. 11754-050), according to the manufacturer's instructions. cDNA was amplified using SsoFast EvaGreen Supermix (Bio-Rad, Hercules, CA, USA, Cat No. 1725201). Real time PCR was performed using gene specific primers for CD14, BCL2, and MCL1. (CD14 Forward Primer: 5' – TCT CTG TCC CCA CAA GTT CC – 3', CD14 Reverse Primer: 5' – CCC GTC CAG TGT CAG GTT ATC – 3', BCL2 Forward Primer: 5' – GCTACC GTC GTG ACT TCG C-3', BCL2 Reverse Primer: 5'- CCC CAC CGA ACT CAA AGA AGG - 3'. MCL1 Forward Primer: 5'- ATG CTT CGG AAA CTG GAC AT - 3', MCL1 Reverse Primer: 5'- TCC TGA TGC CAC CTT CTA GG - 3', GAPDH Forward Primer: 5' – CGACCACTTTGTCAAGCTCA – 3', GAPDH Reverse Primer: 5' – CCCTGTTGCTGTAGCCAAAT – 3'). mRNA levels were normalized against GAPDH mRNA and the amounts of respective mRNA relative to control were calculated using the ΔΔCT method. Relative mRNA expression was demonstrated as fold expression over the average of control gene expression. The fold change in control treatment was assumed to be 1. Values were presented as $\pm$ SEM. Statistical analysis was conducted using Graph Pad Prism (Version 8.4.2). P values < 0.05 were considered statistically significant.

Western blot analysis: The lysates were centrifuged and the supernatants were collected. Protein concentrations were measured using the Pierce BCA Protein Assay kit (Thermo Fisher Scientific, Ref 23227). Protein samples were mixed with sample loading buffer, denatured for 5 min at 95C, resolved on 4-15% SDS – Mini Protein TGC precast gels (Bio-Rad, Cat No. 456-1083), and transferred to Nitrocellulose Membrane 0.45 uM (Bio-Rad, Cat No. 1620115) by LI-COR western blotting (LI-COR Biosciences, Lincoln, NE, USA). Membranes were incubated in the following primary antibodies: BCL-2 mouse monoclonal antibody (Santa Cruz Biotechnology, Dallas, TX, USA, Cat No. sc-509) and BCL-XL rabbit monoclonal antibody (Cell Signaling Technology, Danvers, MA, USA, Cat No. 2762) and normalized to β-Actin goat monoclonal antibody (LifeSpan Biosciencs, Inc., Seattle, WA, USA, Cat No. LS-B15553-50). Proteins of interest were detected using IRDye 800CW Goat anti-Rabbit (926 32211, Lot No. C90220-05), IRDye 800CW Goat anti-Mouse (925-32210, Lot No C90130-03), and IRDye 680RD Donkey anti-Goat (925-68074, Lot No. C90819-04) secondary antibodies (LI-COR) on a LI-COR Odyssey Fc Imaging System.

ID	Top Disease and Functions	Score	Focus Molecules	Molecules in Network
1	Cardiovascular System Development and Function, Cell Morphology, Cellular Development	56	31	Akt, ALCAM, BBX, BMP3, C8orf44-SGK3/SGK3, CRYBG1, EXOSC3, GREB1, HAPLN1, IKZF4, KMT2E, MITF, Notch, NRP2, OSTF1, PAQR3, PROK2, PROX1, PXDNL, RAB28, RBPJ, Secretase gamma, SEMA6D, SLC45A2, SMARCD3, SMYD3, SOX5, SWI-SNF, TFEC, TWIST1, UPK1B, VEGFC, WDFY2, ZEB1, ZFPM2
2	Cell Morphology, Dermatological Diseases and Conditions, Molecular Transport	45	27	AGK, AGO3, ANK3, ARHGEF10L, C5orf30, CCDC102B, DOK5, ECT2, ERK1/2, EXOC5, Fgf, FGF14, FRZB, FXN, Hat, HPSE, IFRD1, ITGB6, KAT6B, MTUS1, N-Cadherin, NELL2, Par6, PARD3, peroxidase (miscellaneous), PEX5L, PHF21A, RIT2, SCN2A, SLC8A1, SPTLC1, TMCC1, voltage-gated sodium channel, WDR7, Wnt
3	Cancer, Organismal Injury and Abnormalities, Respiratory Disease	34	22	14-3-3, ALK, ALKAL1, CASS4, CD3, CDC42SE2, Collagen Alpha1, DDR2, EPHA6, FHAD1, FHIT, GBP1, GTF2IRD1, Hsp27, Hsp90, ICAM5, Jnk, KPNA7, LNPEP, MAP2K6, Mek, MET, Nfat (family), OSBPL8, PACRG, PDGFRA, PFDN4, PTK, R3HDM2, Ral, Ras homolog, TCF, TCR, TPR, ZC3HC1
4	Carbohydrate Metabolism, Cellular Assembly and Organization, Cellular Compromise	32	21	Alpha catenin, ANAPC4, ANAPC7, APC (complex), BIRC2, BPI, CDC23, CFHR5, CLIP2, Cyclin A, Cyclin E, E3 RING, EVI5, F Actin, FOXP2, GLRB, GTPase, HISTONE, Ige, KDM6B, LIMK2, LIMS1, mir-204, mir-31, NFkB (complex), ORC2, PLC gamma, Profilin, PTX3, ROCK1, SYK/ZAP, TNIP3, Ubiquitin, USP17L2 (includes others), WIPF1
5	Cellular Development, Cellular Growth and Proliferation, Embryonic Development	32	21	ATE1, CDK5RAP2, CEP135, CLIP4, CNOT6L, CYP3A7, DHRS3, EBF1, GFI1, GLI3, HDL, HDL-cholesterol, hemoglobin, IgG, IgG1, Igm, Immunoglobulin, N-cor, Nr1h, PARP, PAX5, PHKB, PI3K (family), Pka catalytic subunit, Pkc(s), POLQ, PRDM5, RGMB, Rxr, SPPL2A, SUCLA2, TBC1D19, TTC39B, TTN, UGT2B4
6	Amino Acid Metabolism, Dermatological Diseases and Conditions, Small Molecule Biochemistry	30	20	ABCC1, ADAMTS3, Alp, ATOH1, CHRNA7, COL24A1, collagen, Collagen type I (complex), collagen type i (family), Collagen type II, Collagen type IV, Collagen(s), CXCL13, CXCL5, DLC1, Growth hormone, IL1, KERA, LDL, LGALS3, PDE4D, PI3K (complex), PLOD2, PRLR, RIMS2, SKAP2, SLC1A3, SMAP1, STARD4, STAT5a/b, Tgf beta, TGFBR1, TLL1, Tnf (family), trypsin
7	Cell Morphology, Cell-To-Cell Signaling and Interaction, Nervous System Development and Function	28	19	APBA1, ARHGEF26, CACNA1A, CaMKII, DGKB, Dlg, DLG1, DLG2, ERK, FMN1, GRID2, growth factor receptor, MAGI3, Mlc, NADPH oxidase, NOX4, NTRK3, p70 S6k, Pdgf (complex), PDGF BB, PI3K p85, PLCL1, PP1 protein complex group, Pp1c, Rap1, RBP2, RFC5, RIMBP2, RIMS1, ROBO1, SLIT1, Sos, Syntaxin, THEMIS, TSH
8	Cancer, Cellular Assembly and Organization, Cellular Function and Maintenance	22	16	ALKBH3, AMPK, Ap1, AP1S3, CG, Creb, CREB5, DNAH11, DPT, FSH, HBP1, Hdac, Histone h3, Hsp70, HULC, KCNC2, KIF5B, Lh, LRP1B, LRRN3, Mapk, MEIS2, NMDA Receptor, NRROS, P-TEFb, P38 MAPK, PALLD, Pka, PP2A, Pro-inflammatory Cytokine, RAS, RNA polymerase II, STEAP1, SUN1, Vegf
9	Connective Tissue Disorders, Developmental Disorder, Neurological Disease	22	16	26s Proteasome, Actin, Calmodulin, calpain, caspase, CDH19, CENPU, CUL9, CYB5A, estrogen receptor, Focal adhesion kinase, G6PC2, Gsk3, GSTK1, Histone h4, Insulin, Interferon alpha, IVNS1ABP, Mmp, MTORC1, NCAPG, NPAS3, p85 (pik3r), PCSK2, PLC, PLCB4, Proinsulin, PSMB1, PSMC6, RABGAP1L, Rac, RFTN2, Sfk, SRC (family), TRPM6
10	Cell Cycle, Cellular Growth and Proliferation, Nervous System Development and Function	22	16	7beta-hydroxycholesterol, APP, ARL5A, ARL6IP6, BOD1, CFAP299, DZIP1L, FOS, FSD1L, FZD1, GPR15, GPR3, GPR6, GPR61, GPR78, GPR85, HCFC1, HCFC1R1, KCNIP4, KCNIP4-IT1, KCNMB2, LPAR3, NPS, NTM, platelet activating factor-C16, Plpp1, PRUNE2, RXFP3, SHISA6, SLURP1, SPATA4, SRARP, THAP3, THAP6, ZADH2

Supplementary Table 1: Gene pathways that are associated with ATAC-sequencing peaks in the *ASXL1* mutant cells.

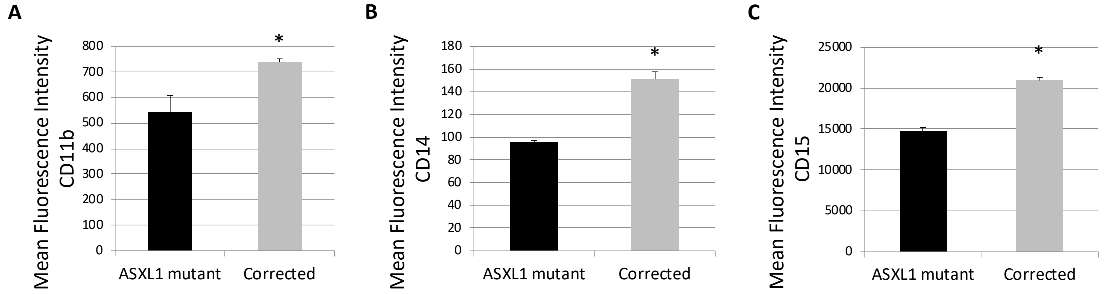

Supplementary Figure 1: Correction of *ASXL1* mutation leads to increase in myeloid differentiation markers: Flow cytometry demonstrates increased mean fluorescence intensity of three myeloid surface markers, CD11b, CD14, and CD15, in the corrected cell line compared to the *ASXL1*^{G710X} mutant cells (Means +/- Stdev, Ttest<0.05, N=3)

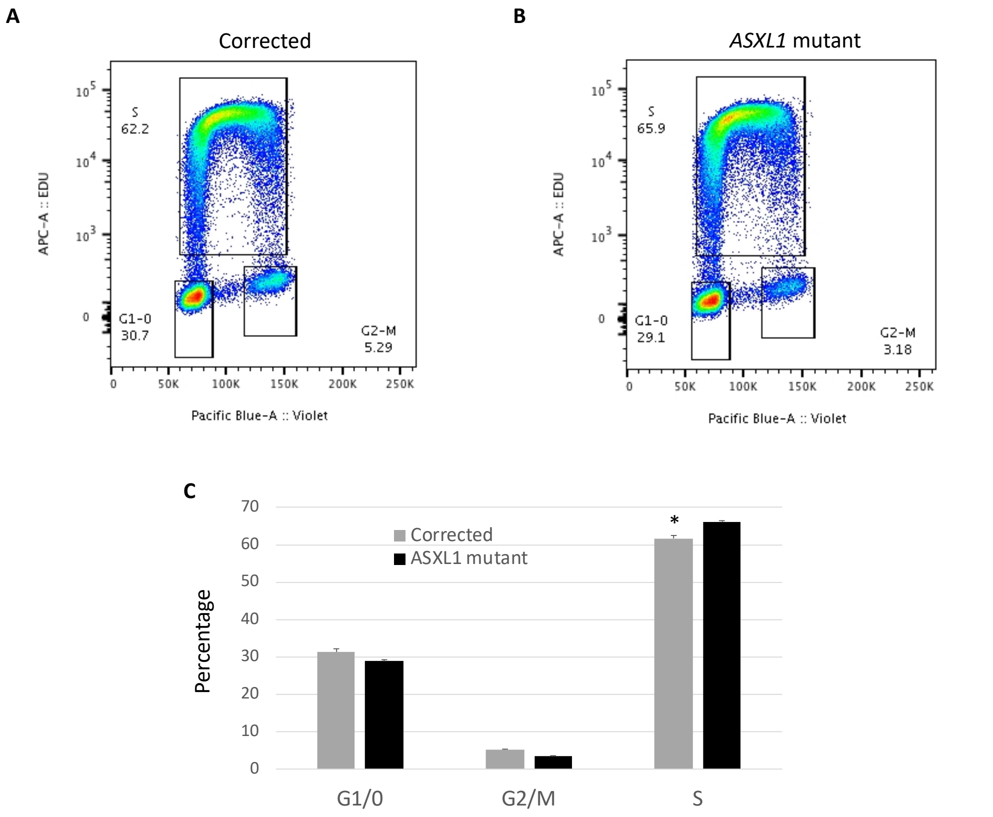

Supplementary Figure 2: Correction of *ASXL1* mutation leads to decrease in S and G2/M cell cycling: Flow cytometry demonstrates decreased proportion of cells in S phase of cell cycle in the corrected cell line compared to the *ASXL1*^{G710X} mutant cells (C). Representative plots are shown (A,B). (Ttest<0.05, N=2)

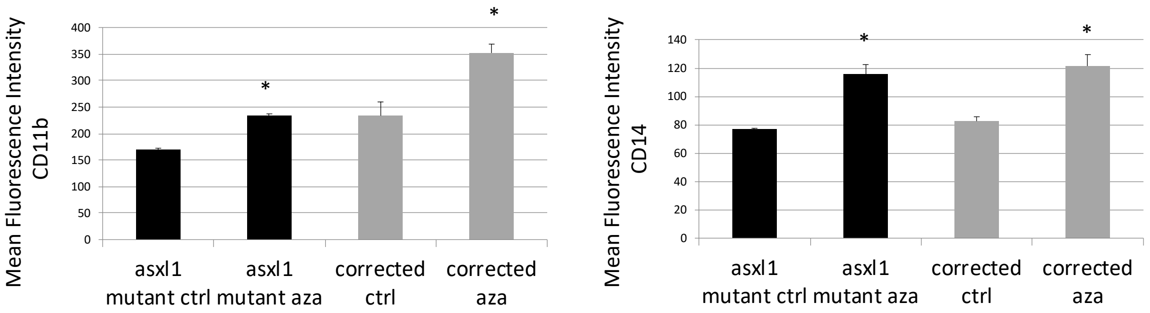

Supplementary Figure 3: Treatment with Azacytidine leads to increase in myeloid differentiation markers: Flow cytometry demonstrates increased mean fluorescence intensity of myeloid surface markers, CD11b and CD14, after treatment with Azacytidine in the corrected cell line and the *ASXL1*^{G710X} mutant cells (Means +/- Stdev, Ttest<0.05, N=3)
